# 3-Methyl-1-phenyl-4-[(*Z*)-phen­yl(4-acetamido­anilino)methyl­idene]-1*H*-pyrazol-5(4*H*)-one

**DOI:** 10.1107/S1600536812026840

**Published:** 2012-06-20

**Authors:** Li-Hua Zhi, Yuan Wang

**Affiliations:** a Department of Physics and Chemistry, Henan Polytechnic University, Jiaozuo 454000, People’s Republic of China

## Abstract

In the title compound, C_25_H_22_N_4_O_2_, the dihedral angles between the central pyrazole ring and the phenyl and benzene rings are 37.01 (3), 75.58 (7) and 49.67 (8)°. An intra­molecular N—H⋯O hydrogen bond generates an *S*(6) motif. In the crystal, N—H⋯O hydrogen bonds link mol­ecules into a zigzag chain extended along the *b* axis.

## Related literature

For the synthesis of Schiff bases derived from 1-phenyl-3-methyl-4-benzoyl-5-pyrazolone and the DNA binding properties of their transition metal complexes, see: Wang & Yang (2005 ▶). For the structure of (*E*,*E*)-3,3′-dimethyl-1,1′-diphenyl-4,4′-{[3-aza­pentane-1,5-diylbis(aza­nedi­yl)]-bis­(phenyl­methyl­idyne)}di-1*H*-pyrazol-5(4*H*)-one, see: Zhang *et al.* (2010 ▶). 
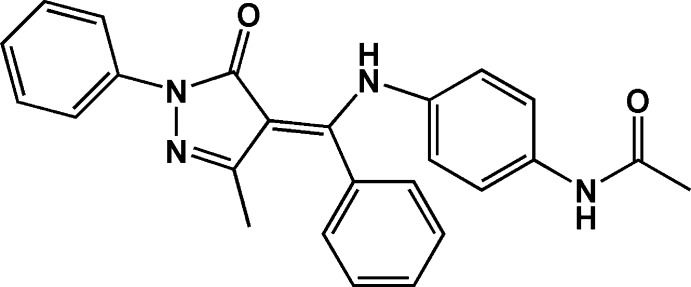


## Experimental

### 

#### Crystal data

C_25_H_22_N_4_O_2_
*M* *_r_* = 410.47
Monoclinic, 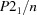

*a* = 7.1800 (4) Å
*b* = 11.0562 (7) Å
*c* = 27.3932 (16) Å
β = 95.138 (4)°
*V* = 2165.8 (2) Å^3^
*Z* = 4
Mo *K*α radiation
μ = 0.08 mm^−1^
*T* = 296 K
0.19 × 0.18 × 0.15 mm

#### Data collection

Bruker APEXII CCD diffractometer
Absorption correction: multi-scan (*SADABS*; Bruker, 2007 ▶) *T*_min_ = 0.985, *T*_max_ = 0.988
19323 measured reflections
5168 independent reflections
3047 reflections with *I* > 2σ(*I*)
*R*_int_ = 0.037

#### Refinement

*R*[*F* ^2^ > 2σ(*F* ^2^)] = 0.053
*wR*(*F* ^2^) = 0.169
*S* = 1.02
5168 reflections
280 parameters
H-atom parameters constrained
Δρ_max_ = 0.18 e Å^−3^
Δρ_min_ = −0.22 e Å^−3^

### 

Data collection: *APEX2* (Bruker, 2007 ▶); cell refinement: *SAINT* (Bruker, 2007 ▶); data reduction: *SAINT*; program(s) used to solve structure: *SHELXS97* (Sheldrick, 2008 ▶); program(s) used to refine structure: *SHELXL97* (Sheldrick, 2008 ▶); molecular graphics: *SHELXTL* (Sheldrick, 2008 ▶); software used to prepare material for publication: *SHELXTL*.

## Supplementary Material

Crystal structure: contains datablock(s) I, global. DOI: 10.1107/S1600536812026840/gk2501sup1.cif

Structure factors: contains datablock(s) I. DOI: 10.1107/S1600536812026840/gk2501Isup2.hkl

Supplementary material file. DOI: 10.1107/S1600536812026840/gk2501Isup3.cml

Additional supplementary materials:  crystallographic information; 3D view; checkCIF report

## Figures and Tables

**Table 1:** Hydrogen-bond geometry (Å, °)

*D*—H⋯*A*	*D*—H	H⋯*A*	*D*⋯*A*	*D*—H⋯*A*
N4—H4*A*⋯O1^i^	0.86	2.05	2.874 (2)	161
N3—H3*A*⋯O1	0.86	1.96	2.696 (2)	143

Symmetry code: (i) 
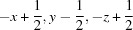
.

**Fig. 1.**
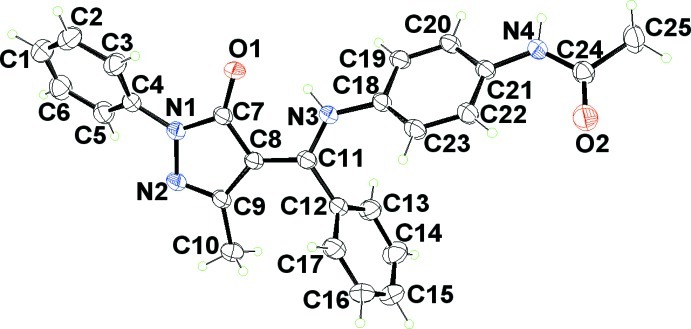
The molecular structure of the title compound shown with 30% probability displacement ellipsoids.

**Fig. 2.**
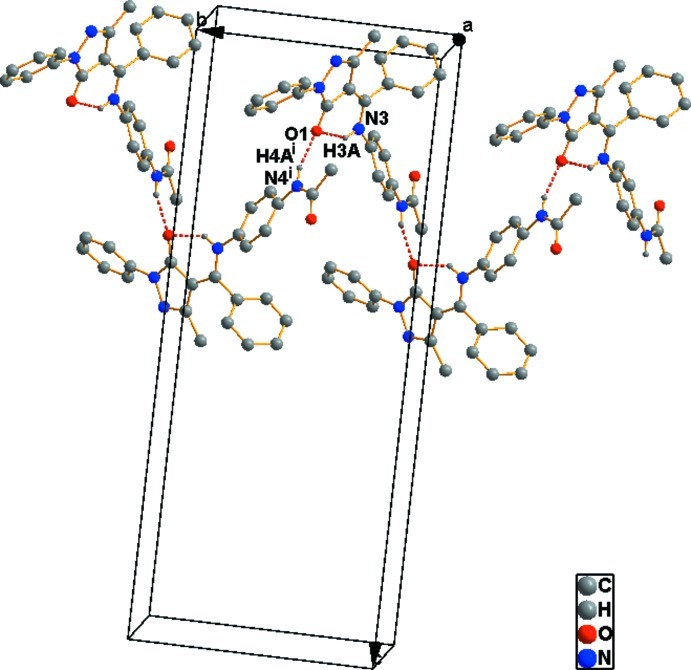
Extended zigzag chain structure along the b axis formed by N—H···O hydrogen bonds [ symmetry code (i): 0.5 - x, y - 0.5, 0.5 - z]. Hydrogen bonds are shown as dashed lines and C-bound H atoms are omitted for clarity.

## supplementary crystallographic information

### Comment

For our interest in coordination chemistry of the Schiff bases derived from 1-phenyl-3-methyl-4-benzoyl-5-pyrazolone (PMBP) (Wang & Yang, 2005; Zhang *et al.*, 2010), the crystal structure of the title compound was determined by X-ray diffraction analysis.

As shown in Fig. 1, in the title molecule , the dihedral angles between the central pyrazole ring (r.m.s. deviation = 0.0094 Å) and the other three benzene rings (C1—C6, r.m.s. deviation = 0.0047 Å, C12—C17, r.m.s. deviation = 0.0097 Å and C18—C23, r.m.s. deviation = 0.0033 Å) are 37.01 (3)°, 75.58 (7)° and 49.67 (8)°, respectively. A strong intramolecular N3—H3···O1 hydrogen bond forms a six-membered ring, producing a S(6) ring motif. In the crystal, intermolecular N—H···O hydrogen bonds link the molecules into a zigzag chain structure along the *b* axis (Table 1, Fig. 2).

### Experimental

1-Phenyl-3-methyl-4-benzoyl-5-pyrazolone (1.1 g, 4 mmol) was dissolved in EtOH (50 ml), and ethanolic solution (10 ml) containing *N*-(4-aminophenyl)acetylamide (0.6 g, 4 mmol) was added dropwise. The reaction mixture was refluxed on a water bath for 3 h, then cooled to room temperature. Yellow block crystals were obtained by slow evaporation of the reaction mixture.

### Refinement

All H atoms were placed in calculated positions, with the carrier atom-H distances = 0.93 Å for aryl, 0.96 Å for methyl and 0.86 Å for the secondary amine H atoms, and refined as riding, with the *U*_iso_(H) = 1.5U_eq_(C) for methyl groups and 1.2U_eq_(C,*N*) for others.

### Crystal data

**Table:**

C_25_H_22_N_4_O_2_	*F*(000) = 864
*M**_r_* = 410.47	*D*_x_ = 1.259 Mg m^−^^3^
Monoclinic, *P*2_1_/*n*	Melting point: 567(9) K
Hall symbol: -P 2yn	Mo *K*α radiation, λ = 0.71073 Å
*a* = 7.1800 (4) Å	Cell parameters from 3801 reflections
*b* = 11.0562 (7) Å	θ = 2.4–22.2°
*c* = 27.3932 (16) Å	µ = 0.08 mm^−^^1^
β = 95.138 (4)°	*T* = 296 K
*V* = 2165.8 (2) Å^3^	Block, yellow
*Z* = 4	0.19 × 0.18 × 0.15 mm

### Data collection

**Table:**

Bruker APEXII CCD diffractometer	5168 independent reflections
Radiation source: fine-focus sealed tube	3047 reflections with *I* > 2σ(*I*)
Graphite monochromator	*R*_int_ = 0.037
φ and ω scans	θ_max_ = 27.9°, θ_min_ = 1.5°
Absorption correction: multi-scan (*SADABS*; Bruker, 2007)	*h* = −9→9
*T*_min_ = 0.985, *T*_max_ = 0.988	*k* = −14→12
19323 measured reflections	*l* = −36→31

### Refinement

**Table:**

Refinement on *F*^2^	Primary atom site location: structure-invariant direct methods
Least-squares matrix: full	Secondary atom site location: difference Fourier map
*R*[*F*^2^ > 2σ(*F*^2^)] = 0.053	Hydrogen site location: inferred from neighbouring sites
*wR*(*F*^2^) = 0.169	H-atom parameters constrained
*S* = 1.02	*w* = 1/[σ^2^(*F*_o_^2^) + (0.0834*P*)^2^ + 0.3021*P*] where *P* = (*F*_o_^2^ + 2*F*_c_^2^)/3
5168 reflections	(Δ/σ)_max_ < 0.001
280 parameters	Δρ_max_ = 0.18 e Å^−^^3^
0 restraints	Δρ_min_ = −0.22 e Å^−^^3^

### Special details

**Table:**

Geometry. All e.s.d.'s (except the e.s.d. in the dihedral angle between two l.s. planes) are estimated using the full covariance matrix. The cell e.s.d.'s are taken into account individually in the estimation of e.s.d.'s in distances, angles and torsion angles; correlations between e.s.d.'s in cell parameters are only used when they are defined by crystal symmetry. An approximate (isotropic) treatment of cell e.s.d.'s is used for estimating e.s.d.'s involving l.s. planes.
Refinement. Refinement of *F*^2^ against ALL reflections. The weighted *R*-factor *wR* and goodness of fit *S* are based on *F*^2^, conventional *R*-factors *R* are based on *F*, with *F* set to zero for negative *F*^2^. The threshold expression of *F*^2^ > σ(*F*^2^) is used only for calculating *R*-factors(gt) *etc*. and is not relevant to the choice of reflections for refinement. *R*-factors based on *F*^2^ are statistically about twice as large as those based on *F*, and *R*- factors based on ALL data will be even larger.

### Fractional atomic coordinates and isotropic or equivalent isotropic displacement parameters (Å^2^)

**Table:**

	*x*	*y*	*z*	*U*_iso_*/*U*_eq_	
C1	1.1453 (4)	0.8096 (3)	0.15116 (9)	0.0775 (7)	
H1A	1.2285	0.8712	0.1610	0.093*	
C2	0.9601 (4)	0.8167 (2)	0.16072 (10)	0.0790 (7)	
H2B	0.9189	0.8829	0.1777	0.095*	
C3	0.8358 (3)	0.7268 (2)	0.14534 (8)	0.0648 (6)	
H3B	0.7110	0.7325	0.1517	0.078*	
C4	0.8971 (3)	0.62867 (18)	0.12057 (7)	0.0485 (5)	
C5	1.0823 (3)	0.6204 (2)	0.11169 (8)	0.0600 (6)	
H5A	1.1247	0.5535	0.0953	0.072*	
C6	1.2045 (3)	0.7111 (2)	0.12710 (9)	0.0718 (7)	
H6A	1.3295	0.7051	0.1210	0.086*	
C7	0.6243 (3)	0.48691 (17)	0.12369 (7)	0.0464 (5)	
C8	0.5483 (3)	0.39817 (17)	0.08929 (6)	0.0441 (5)	
C9	0.6660 (3)	0.40227 (18)	0.04957 (7)	0.0498 (5)	
C10	0.6607 (4)	0.3296 (2)	0.00387 (8)	0.0725 (7)	
H10A	0.7596	0.3552	−0.0151	0.109*	
H10B	0.5424	0.3410	−0.0148	0.109*	
H10C	0.6766	0.2456	0.0121	0.109*	
C11	0.4038 (3)	0.31978 (17)	0.09954 (6)	0.0447 (4)	
C12	0.3277 (3)	0.22780 (18)	0.06380 (6)	0.0456 (5)	
C13	0.3630 (3)	0.10682 (19)	0.07272 (8)	0.0581 (6)	
H13A	0.4253	0.0827	0.1023	0.070*	
C14	0.3063 (4)	0.0220 (2)	0.03798 (9)	0.0746 (7)	
H14A	0.3336	−0.0593	0.0438	0.090*	
C15	0.2096 (4)	0.0564 (3)	−0.00520 (9)	0.0771 (7)	
H15A	0.1720	−0.0014	−0.0287	0.092*	
C16	0.1685 (4)	0.1755 (3)	−0.01361 (8)	0.0719 (7)	
H16A	0.0998	0.1984	−0.0425	0.086*	
C17	0.2282 (3)	0.2620 (2)	0.02036 (7)	0.0617 (6)	
H17A	0.2018	0.3432	0.0142	0.074*	
C18	0.1978 (3)	0.25908 (17)	0.16386 (7)	0.0463 (5)	
C19	0.2328 (3)	0.21907 (19)	0.21160 (7)	0.0498 (5)	
H19A	0.3489	0.2333	0.2285	0.060*	
C20	0.0977 (3)	0.15856 (18)	0.23422 (7)	0.0491 (5)	
H20A	0.1231	0.1323	0.2664	0.059*	
C21	−0.0763 (3)	0.13596 (17)	0.20977 (6)	0.0443 (4)	
C22	−0.1115 (3)	0.17695 (19)	0.16203 (7)	0.0549 (5)	
H22A	−0.2276	0.1631	0.1451	0.066*	
C23	0.0252 (3)	0.2384 (2)	0.13947 (7)	0.0550 (5)	
H23A	−0.0001	0.2660	0.1075	0.066*	
C24	−0.3715 (3)	0.02447 (19)	0.21917 (8)	0.0542 (5)	
C25	−0.4773 (3)	−0.0323 (2)	0.25790 (9)	0.0727 (7)	
H25A	−0.5909	−0.0673	0.2430	0.109*	
H25B	−0.5068	0.0283	0.2811	0.109*	
H25C	−0.4021	−0.0942	0.2744	0.109*	
O1	0.57427 (19)	0.51393 (13)	0.16510 (5)	0.0552 (4)	
O2	−0.4273 (2)	0.02170 (19)	0.17623 (6)	0.0864 (6)	
N1	0.7704 (2)	0.53743 (15)	0.10245 (6)	0.0514 (4)	
N2	0.7976 (2)	0.48311 (15)	0.05741 (6)	0.0551 (5)	
N3	0.3406 (2)	0.32684 (15)	0.14348 (5)	0.0518 (4)	
H3A	0.3941	0.3806	0.1626	0.062*	
N4	−0.2087 (2)	0.07663 (15)	0.23616 (5)	0.0512 (4)	
H4A	−0.1822	0.0732	0.2674	0.061*	

### Atomic displacement parameters (Å^2^)

**Table:**

	*U*^11^	*U*^22^	*U*^33^	*U*^12^	*U*^13^	*U*^23^
C1	0.0842 (18)	0.0814 (18)	0.0656 (15)	−0.0301 (15)	−0.0001 (13)	−0.0072 (13)
C2	0.0926 (19)	0.0665 (16)	0.0795 (16)	−0.0165 (14)	0.0161 (14)	−0.0244 (13)
C3	0.0673 (14)	0.0582 (14)	0.0711 (14)	−0.0075 (11)	0.0183 (11)	−0.0156 (11)
C4	0.0577 (12)	0.0457 (12)	0.0431 (10)	−0.0046 (9)	0.0104 (9)	0.0010 (8)
C5	0.0606 (13)	0.0569 (14)	0.0638 (13)	−0.0006 (11)	0.0125 (11)	0.0008 (10)
C6	0.0555 (14)	0.0841 (18)	0.0753 (15)	−0.0111 (13)	0.0033 (12)	0.0061 (14)
C7	0.0548 (11)	0.0453 (11)	0.0406 (9)	0.0002 (9)	0.0130 (8)	0.0001 (8)
C8	0.0556 (11)	0.0415 (11)	0.0364 (9)	−0.0023 (9)	0.0103 (8)	−0.0021 (8)
C9	0.0648 (13)	0.0457 (11)	0.0412 (10)	−0.0044 (10)	0.0174 (9)	−0.0013 (8)
C10	0.0918 (17)	0.0774 (17)	0.0527 (12)	−0.0174 (14)	0.0322 (12)	−0.0193 (11)
C11	0.0527 (11)	0.0445 (11)	0.0376 (9)	0.0017 (9)	0.0076 (8)	0.0018 (8)
C12	0.0535 (11)	0.0477 (11)	0.0366 (9)	−0.0034 (9)	0.0103 (8)	0.0011 (8)
C13	0.0704 (14)	0.0509 (13)	0.0518 (11)	0.0013 (11)	−0.0013 (10)	0.0011 (10)
C14	0.0971 (19)	0.0516 (14)	0.0741 (16)	−0.0021 (13)	0.0011 (14)	−0.0119 (12)
C15	0.0907 (18)	0.0791 (19)	0.0612 (14)	−0.0218 (15)	0.0061 (13)	−0.0232 (13)
C16	0.0824 (17)	0.090 (2)	0.0408 (11)	−0.0137 (15)	−0.0053 (11)	0.0003 (12)
C17	0.0786 (15)	0.0601 (14)	0.0458 (11)	−0.0037 (12)	0.0027 (10)	0.0091 (10)
C18	0.0537 (12)	0.0461 (11)	0.0410 (10)	−0.0025 (9)	0.0143 (8)	−0.0015 (8)
C19	0.0500 (11)	0.0606 (13)	0.0391 (9)	−0.0010 (9)	0.0068 (8)	−0.0012 (9)
C20	0.0556 (12)	0.0570 (13)	0.0349 (9)	0.0007 (10)	0.0061 (8)	0.0057 (8)
C21	0.0516 (11)	0.0429 (11)	0.0392 (9)	−0.0005 (9)	0.0087 (8)	0.0020 (8)
C22	0.0566 (12)	0.0653 (14)	0.0429 (10)	−0.0089 (11)	0.0052 (9)	0.0079 (9)
C23	0.0600 (13)	0.0674 (14)	0.0375 (10)	−0.0052 (11)	0.0042 (9)	0.0098 (9)
C24	0.0538 (12)	0.0580 (13)	0.0509 (12)	−0.0018 (10)	0.0055 (9)	0.0094 (10)
C25	0.0667 (15)	0.0776 (17)	0.0745 (15)	−0.0185 (13)	0.0091 (12)	0.0209 (13)
O1	0.0633 (9)	0.0631 (9)	0.0418 (7)	−0.0091 (7)	0.0185 (6)	−0.0139 (6)
O2	0.0724 (11)	0.1269 (16)	0.0576 (10)	−0.0295 (11)	−0.0074 (8)	0.0188 (10)
N1	0.0626 (10)	0.0486 (10)	0.0458 (9)	−0.0102 (8)	0.0200 (8)	−0.0080 (7)
N2	0.0714 (11)	0.0540 (10)	0.0429 (9)	−0.0074 (9)	0.0226 (8)	−0.0058 (7)
N3	0.0621 (10)	0.0549 (10)	0.0404 (8)	−0.0136 (8)	0.0161 (7)	−0.0061 (7)
N4	0.0578 (10)	0.0577 (11)	0.0386 (8)	−0.0110 (9)	0.0070 (7)	0.0064 (7)

### Geometric parameters (Å, º)

**Table:**

C1—C6	1.361 (4)	C14—C15	1.371 (4)
C1—C2	1.380 (4)	C14—H14A	0.9300
C1—H1A	0.9300	C15—C16	1.365 (4)
C2—C3	1.376 (3)	C15—H15A	0.9300
C2—H2B	0.9300	C16—C17	1.376 (3)
C3—C4	1.373 (3)	C16—H16A	0.9300
C3—H3B	0.9300	C17—H17A	0.9300
C4—C5	1.376 (3)	C18—C23	1.374 (3)
C4—N1	1.418 (2)	C18—C19	1.382 (3)
C5—C6	1.374 (3)	C18—N3	1.424 (2)
C5—H5A	0.9300	C19—C20	1.371 (3)
C6—H6A	0.9300	C19—H19A	0.9300
C7—O1	1.256 (2)	C20—C21	1.386 (3)
C7—N1	1.364 (2)	C20—H20A	0.9300
C7—C8	1.434 (3)	C21—C22	1.386 (3)
C8—C11	1.399 (3)	C21—N4	1.407 (2)
C8—C9	1.437 (2)	C22—C23	1.384 (3)
C9—N2	1.304 (3)	C22—H22A	0.9300
C9—C10	1.485 (3)	C23—H23A	0.9300
C10—H10A	0.9600	C24—O2	1.209 (3)
C10—H10B	0.9600	C24—N4	1.348 (3)
C10—H10C	0.9600	C24—C25	1.497 (3)
C11—N3	1.326 (2)	C25—H25A	0.9600
C11—C12	1.482 (3)	C25—H25B	0.9600
C12—C13	1.379 (3)	C25—H25C	0.9600
C12—C17	1.385 (3)	N1—N2	1.401 (2)
C13—C14	1.372 (3)	N3—H3A	0.8600
C13—H13A	0.9300	N4—H4A	0.8600
			
C6—C1—C2	119.0 (2)	C16—C15—H15A	120.1
C6—C1—H1A	120.5	C14—C15—H15A	120.1
C2—C1—H1A	120.5	C15—C16—C17	120.5 (2)
C3—C2—C1	120.7 (2)	C15—C16—H16A	119.8
C3—C2—H2B	119.7	C17—C16—H16A	119.8
C1—C2—H2B	119.7	C16—C17—C12	119.8 (2)
C2—C3—C4	119.6 (2)	C16—C17—H17A	120.1
C2—C3—H3B	120.2	C12—C17—H17A	120.1
C4—C3—H3B	120.2	C23—C18—C19	119.12 (17)
C3—C4—C5	119.8 (2)	C23—C18—N3	123.15 (17)
C3—C4—N1	120.75 (18)	C19—C18—N3	117.60 (18)
C5—C4—N1	119.38 (18)	C20—C19—C18	120.51 (19)
C4—C5—C6	119.8 (2)	C20—C19—H19A	119.7
C4—C5—H5A	120.1	C18—C19—H19A	119.7
C6—C5—H5A	120.1	C19—C20—C21	120.86 (17)
C1—C6—C5	121.0 (2)	C19—C20—H20A	119.6
C1—C6—H6A	119.5	C21—C20—H20A	119.6
C5—C6—H6A	119.5	C22—C21—C20	118.52 (17)
O1—C7—N1	125.51 (18)	C22—C21—N4	124.24 (18)
O1—C7—C8	129.31 (17)	C20—C21—N4	117.18 (16)
N1—C7—C8	105.17 (15)	C21—C22—C23	120.34 (19)
C11—C8—C7	122.49 (15)	C21—C22—H22A	119.8
C11—C8—C9	131.94 (17)	C23—C22—H22A	119.8
C7—C8—C9	105.13 (16)	C18—C23—C22	120.64 (18)
N2—C9—C8	111.31 (16)	C18—C23—H23A	119.7
N2—C9—C10	118.23 (16)	C22—C23—H23A	119.7
C8—C9—C10	130.45 (19)	O2—C24—N4	123.28 (18)
C9—C10—H10A	109.5	O2—C24—C25	122.3 (2)
C9—C10—H10B	109.5	N4—C24—C25	114.45 (18)
H10A—C10—H10B	109.5	C24—C25—H25A	109.5
C9—C10—H10C	109.5	C24—C25—H25B	109.5
H10A—C10—H10C	109.5	H25A—C25—H25B	109.5
H10B—C10—H10C	109.5	C24—C25—H25C	109.5
N3—C11—C8	117.75 (17)	H25A—C25—H25C	109.5
N3—C11—C12	120.29 (16)	H25B—C25—H25C	109.5
C8—C11—C12	121.91 (15)	C7—N1—N2	111.92 (15)
C13—C12—C17	119.33 (19)	C7—N1—C4	129.48 (15)
C13—C12—C11	119.85 (18)	N2—N1—C4	118.51 (14)
C17—C12—C11	120.75 (18)	C9—N2—N1	106.42 (14)
C14—C13—C12	120.1 (2)	C11—N3—C18	129.94 (17)
C14—C13—H13A	119.9	C11—N3—H3A	115.0
C12—C13—H13A	119.9	C18—N3—H3A	115.0
C15—C14—C13	120.3 (2)	C24—N4—C21	128.81 (16)
C15—C14—H14A	119.8	C24—N4—H4A	115.6
C13—C14—H14A	119.8	C21—N4—H4A	115.6
C16—C15—C14	119.9 (2)		
			
C6—C1—C2—C3	−1.3 (4)	C11—C12—C17—C16	176.10 (19)
C1—C2—C3—C4	0.5 (4)	C23—C18—C19—C20	0.6 (3)
C2—C3—C4—C5	0.6 (3)	N3—C18—C19—C20	176.74 (17)
C2—C3—C4—N1	−177.2 (2)	C18—C19—C20—C21	0.2 (3)
C3—C4—C5—C6	−0.8 (3)	C19—C20—C21—C22	−0.6 (3)
N1—C4—C5—C6	176.98 (19)	C19—C20—C21—N4	−178.09 (18)
C2—C1—C6—C5	1.0 (4)	C20—C21—C22—C23	0.3 (3)
C4—C5—C6—C1	0.1 (4)	N4—C21—C22—C23	177.62 (18)
O1—C7—C8—C11	−3.9 (3)	C19—C18—C23—C22	−0.9 (3)
N1—C7—C8—C11	174.95 (18)	N3—C18—C23—C22	−176.79 (18)
O1—C7—C8—C9	−177.1 (2)	C21—C22—C23—C18	0.4 (3)
N1—C7—C8—C9	1.7 (2)	O1—C7—N1—N2	176.45 (19)
C11—C8—C9—N2	−172.8 (2)	C8—C7—N1—N2	−2.4 (2)
C7—C8—C9—N2	−0.5 (2)	O1—C7—N1—C4	0.0 (3)
C11—C8—C9—C10	6.0 (4)	C8—C7—N1—C4	−178.89 (19)
C7—C8—C9—C10	178.3 (2)	C3—C4—N1—C7	−40.4 (3)
C7—C8—C11—N3	−2.1 (3)	C5—C4—N1—C7	141.8 (2)
C9—C8—C11—N3	169.1 (2)	C3—C4—N1—N2	143.3 (2)
C7—C8—C11—C12	−179.45 (18)	C5—C4—N1—N2	−34.5 (3)
C9—C8—C11—C12	−8.3 (3)	C8—C9—N2—N1	−1.0 (2)
N3—C11—C12—C13	−67.8 (3)	C10—C9—N2—N1	−179.88 (19)
C8—C11—C12—C13	109.5 (2)	C7—N1—N2—C9	2.2 (2)
N3—C11—C12—C17	115.0 (2)	C4—N1—N2—C9	179.07 (18)
C8—C11—C12—C17	−67.7 (3)	C8—C11—N3—C18	−179.58 (19)
C17—C12—C13—C14	2.6 (3)	C12—C11—N3—C18	−2.2 (3)
C11—C12—C13—C14	−174.6 (2)	C23—C18—N3—C11	−47.3 (3)
C12—C13—C14—C15	−1.9 (4)	C19—C18—N3—C11	136.7 (2)
C13—C14—C15—C16	−0.3 (4)	O2—C24—N4—C21	0.3 (4)
C14—C15—C16—C17	1.9 (4)	C25—C24—N4—C21	179.2 (2)
C15—C16—C17—C12	−1.2 (4)	C22—C21—N4—C24	16.4 (3)
C13—C12—C17—C16	−1.1 (3)	C20—C21—N4—C24	−166.3 (2)

### Hydrogen-bond geometry (Å, º)

**Table:**

*D*—H···*A*	*D*—H	H···*A*	*D*···*A*	*D*—H···*A*
N4—H4*A*···O1^i^	0.86	2.05	2.874 (2)	161
N3—H3*A*···O1	0.86	1.96	2.696 (2)	143

Symmetry code: (i) −*x*+1/2, *y*−1/2, −*z*+1/2.
